# Enabling Real-Time Quality-of-Service and Fine-Grained Aggregation for Wireless TSN

**DOI:** 10.3390/s22103901

**Published:** 2022-05-20

**Authors:** Litianyi Zhang, Yifan Gu, Rui Wang, Kan Yu, Zhibo Pang, Yonghui Li, Branka Vucetic

**Affiliations:** 1 School of Electrical and Information Engineering, The University of Sydney, Sydney, NSW 2006, Australia; litianyi.zhang@sydney.edu.au (L.Z.); r.wang@sydney.edu.au (R.W.); yonghui.li@sydney.edu.au (Y.L.); branka.vucetic@sydney.edu.au (B.V.); 2 Department of Computer Science and Information Technology, La Trobe University, Bendigo, VIC 3552, Australia; k.yu@latrobe.edu.au; 3 Department of Automation Technology, ABB Corporate Research Sweden, 72178 Vasteras, Sweden; zhibo@kth.se; 4 Department of Intelligent Systems, Royal Institute of Technology (KTH), 10044 Stockholm, Sweden

**Keywords:** wireless time-sensitive networking, industrial IoT, IEEE 802.11

## Abstract

Wireless Time-Sensitive Networking (WTSN) has emerged as a promising technology for Industrial Internet of Things (IIoT) applications. To meet the latency requirements of WTSN, wireless local area network (WLAN) such as IEEE 802.11 protocol with the time division multiple access (TDMA) mechanism is shown to be a practical solution. In this paper, we propose the RT-WiFiQA protocol with two novel schemes to improve the latency and reliability performance: real-time quality of service (RT-QoS) and fine-grained aggregation (FGA) for TDMA-based 802.11 systems. The RT-QoS is designed to guarantee the quality-of-service requirements of different traffic and to support the FGA mechanism. The FGA mechanism aggregates frames for different stations to reduce the physical layer transmission overhead. The trade-off between the reliability and FGA packet size is analyzed with numerical results. Specifically, we derive a critical threshold such that the FGA can achieve higher reliability when the aggregated packet size is smaller than the critical threshold. Otherwise, the non-aggregation scheme outperforms the FGA scheme. Extensive experiments are conducted on the commercial off-the-shelf 802.11 interface. The experiment results show that compared with the existing TDMA-based 802.11 system, the developed RT-WiFiQA protocol can achieve deterministic bounded real-time latency and greatly improves the reliability performance.

## 1. Introduction

With the increasing demand for unmanned devices and automatic control systems, the Industrial Internet of Things (IIoT) has attracted significant attention and become one of the most critical aspects in Industry 4.0 [1]. Different from the conventional Internet of Things (IoT) applications, IIoT applications have very stringent requirements in terms of precise synchronization, transmission reliability, and bounded latency. Time-Sensitive Networking (TSN), proposed by IEEE 802.1 TSN Task Group [2], is a promising solution to meet the stringent requirements of IIoT by utilizing the collision-free and low packet error rate (PER) features of wired connections. However, compared with wired communication solutions, wireless technologies are flexible and scalable, and can be deployed easily and rapidly. The development of enabling wireless TSN (WTSN) for IIoT has recently attracted much attention [3]. It is challenging for wireless technologies to meet the stringent latency and reliability requirements of critical IIoT applications due to the shared medium and collision environment of wireless channels.

As one of the most widely applied wireless protocols [4], IEEE 802.11 WiFi systems can achieve high-rate transmissions and potentially meet the stringent latency requirements of IIoT applications by optimizing their algorithms and protocols. The existing WiFi technologies adopt carrier sense multiple access with collision avoidance (CSMA/CA) mechanism with distributed random access, which cannot guarantee deterministic latency and reliability. Therefore, some research work focused on the modification of the legacy 802.11 medium access control (MAC) layer towards the WTSN. For example, RT-WiFi [5], Soft-TDMAC [6], and Det-WiFi [7] were proposed based on the time division multiple access (TDMA) protocol and implemented on the IEEE 802.11 commercial off-the-shelf (COTS) network interfaces. However, these designs mainly focused on reducing the latency without optimizing the transmission efficiency and reliability. Moreover, such existing works only consider a single traffic type. How to design an effective system for multiple traffic types to meet their respective quality of service (QoS) requirements in IIoT applications remains an open problem.

In this paper, we propose the real-time WiFi protocol with QoS and aggregation (RT-WiFiQA) by introducing two novel schemes to enhance the performance of the TDMA-based 802.11 systems: real-time quality of service (RT-QoS) and fine-grained aggregation (FGA). We realize that it can be hard to develop a rigid design for a wide range of IIoT applications because different applications may have significantly different requirements of reliability, latency, packet generation rates, etc. Therefore, we aim at providing a flexible and transparent design such that we create user application programming interfaces (APIs) for the settings of the proposed RT-WiFiQA protocol. The control designers can have great flexibility in choosing their setups in terms of RT-QoS and FGA based on specific application requirements. Furthermore, the design of the RT-WiFiQA protocol is based on the COTS 802.11 interface and compatible with the existing 802.11 applications with no or minimal modifications.

The proposed RT-QoS scheme can accommodate different traffic types, guarantee their QoS requirements, and support the FGA mechanism. For the system with mixed real-time and non-real-time traffic, the conventional periodic time slots allocation cannot meet the QoS requirements of real-time traffic. RT-QoS can optimize the allocation of time slots based on the distributions of traffic types, their QoS requirements, and available time slot resources. Furthermore, by jointly designing the RT-QoS and application (APP)-layer retransmissions, the reliability on the APP layer can be greatly improved.

The proposed FGA can improve the downlink transmission efficiency by significantly reducing the overhead. We also realize that there is a fundamental trade-off in packet aggregation when considering the WTSN. On one hand, it reduces the overhead, thus improving the transmission efficiency and allowing for more retransmissions. On the other hand, it results in a higher PER for each transmission due to a longer packet length. A natural question arises: *Will the packet aggregation scheme benefit the WTSN or not in terms of reliability and latency*? In order to answer this critical question, the trade-off is analyzed, and comprehensive simulations are conducted to validate the impact of the proposed FGA on reliability. We also give insights on how to choose the aggregation parameters for the design of RT-WiFiQA networks according to our analysis. To the best knowledge of the authors, this is the first paper that studies the packet aggregation in WTSN with detailed implementation and trade-off analysis.

The contributions of this work are summarized as follows:

- We propose the RT-WiFiQA protocol with RT-QoS and FGA mechanisms to improve the performance in terms of latency and reliability on the TDMA-based 802.11 system. We also implement the developed schemes on COTS 802.11 interfaces. The detailed implementation with APIs is also provided.
- We analytically show that the FGA mechanism can outperform non-aggregation in terms of latency and reliability when the FGA packet size is smaller than a critical threshold. Numerical simulations are also conducted to validate our theoretical analysis, and the evaluated critical threshold is also applied in the practical FGA implementation.
- We perform extensive experiments to measure the APP-layer and the MAC-layer latency and reliability on our hardware platform. The experimental results demonstrate the superiority of the proposed RT-WiFiQA protocol compared with the existing TDMA-based 802.11 protocol and conventional 802.11 protocol.

## 2. Related Works

Some recent wireless protocols have been carried out to meet the stringent latency and reliability requirements of WTSN. WirelessHP [8] and w-SHARP [9] were proposed based on software-defined radio (SDR) by optimizing both the physical (PHY) and MAC layers to achieve μs-level latency and packet loss ratio lower than $10^{-6}$. However, these solutions have low compatibility with existing wireless standards and are highly costly to be implemented in practical systems. There is also some work improving the MAC layer of the existing IEEE 802.15.4 protocol, such as WirelessHART [10] and ISA100.11a [11]. However, the data rate of IEEE 802.15.4 is only up to 250 kb/s and cannot satisfy the requirements of high rate transmissions in many IIoT applications. In addition, reconfigurable intelligent surface and satellite-terrestrial networks are also investigated to improve the transmission reliability in IIoT networks [12,13]. However, these solutions may require additional hardware equipment, which can increase the cost and system complexity. Recently, 5G Ultra-reliable and Low-latency Communication has been proposed by 3GPP [14,15]. However, the existing TSN is established on the 802 link layers, which is not fully compatible with the 3GPP-based 5G standard [3].

Due to the advantages of compatibility, cost, high rate, etc., many research works focused on the modification of IEEE 802.11 protocols based on the COTS network interfaces towards WTSN. In order to improve the reliability of industrial Wi-Fi networks, the authors in [16] proposed Wi-Fi Redundancy (Wi-Red) solution to offer seamless link-level redundancy. However, each independent Wi-Fi network in Wi-Red still uses legacy CSMA/CA, which cannot guarantee deterministic latency. In [5], the authors proposed the RT-WiFi protocol, which designed a scheduler to allocate a sequence of time slots for each station and can achieve a sampling rate of 6 kHz. The authors in [6] proposed the Soft-TDMAC based on TDMA protocol to improve the synchronization precision. The authors in [7] conducted TDMA scheduling implementation on COTS hardware with support for multi-hop networks, namely Det-WiFi. These protocols utilized TDMA to guarantee the latency without considering efficiency or reliability optimization. Very recently, the authors in [17] designed HAR${}^{2}$D-Fi to provide reliable and deterministic communication based on the latest IEEE 802.11ax protocol. Differently, our work focuses on the implementation of QoS and aggregation mechanisms for WTSN, and the proposed schemes are validated on COTS hardware platforms, while HAR${}^{2}$D-Fi was only validated through simulation.

To improve the transmission efficiency in WTSN, applying aggregation schemes can be an effective and promising solution. The conventional wired TSN proposed aggregation schemes, namely the Link Aggregation Control Protocol [18], but it cannot be extended to WTSN directly because of the error-prone features of wireless channels. In 802.11n, the A-MPDU and A-MSDU schemes can aggregate packets towards a single destination and are designed for throughput maximization. However, the A-MPDU and A-MSDU schemes cannot achieve low latency because of the time consumption for generating the aggregated packet [19]. Moreover, the packet aggregation mechanism proposed for WirelessHART focused on the 802.15.4 adhoc mode and cannot be implemented on the considered 802.11 infrastructure mode [20,21,22]. Additionally, WIA-FA proposed a similar aggregation mechanism for 802.11 interfaces [23]. However, the detailed implementation with APIs is not designed and discussed. Moreover, the aforementioned critical trade-off in determining whether to use aggregation is not analyzed.

## 3. System Design and Implementation

**Figure 1:**
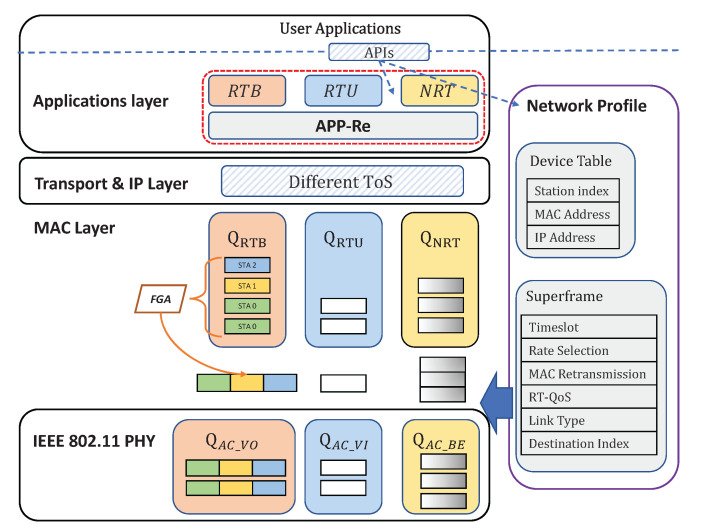
Overview of framework architecture for RT-WiFiQA.

The architecture of our proposed RT-WiFiQA protocol is shown in Figure 1. The user applications represent a group of concurrent applications with various timeliness, sampling rates, and reliability requirements. We provide APIs for users to allocate an RT-QoS value and determine a specific traffic setting for each packet in terms of its QoS requirements. The RT-QoS is designed to guarantee the QoS requirements of real-time traffic and enable the FGA scheme, which is explained in detail in Section 3.1. The FGA scheme aims to reduce the overhead and improve the downlink transmission reliability and efficiency, which is introduced in detail in Section 3.2. Besides the two enhancements, we also provide APIs for traffic settings, including the APP-layer retransmission (APP-Re) and the network profile. Lastly, for the basic TDMA system, we follow the design proposed in [5] for COTS 802.11 interfaces that can achieve a synchronization accuracy of 20 μs and a time slot duration as low as around 100 μs.

### 3.1. APP-Layer Configuration and RT-QoS

Basic MAC-layer retransmissions of the TDMA-based 802.11 system are conducted within one time slot, which is executed if the sender does not receive the acknowledgment (ACK) packet from the receiver. However, in the TDMA-based IIoT system without carrier sense, the MAC-layer retransmissions may fail when burst interference exists. Therefore, we develop APIs for the APP-Re scheme, which can efficiently avoid the burst interference by retransmitting the packets at different time instants. The procedure of APP-Re is addressed as follows. Once senders start a transmission event, each packet is allocated with a unique sequence number. Receivers will reply with an APP-layer ACK to the sender when receiving a new packet and record the sequence number at the same time. Senders stop retransmissions once the ACK is received. Otherwise, the APP-Re keeps executing until the maximum retransmission time limit is reached. Receivers drop packets with the same recorded sequence. Besides the APP-Re solution, other error control coding techniques can also be added according to the application specific requirements through our APIs.

The network file is a static file to control the transmission pattern and to maintain the network information, including a device table and a superframe structure maintained by the access point (AP) and all stations. The device table contains the MAC address, IP address, and a unique index of each station. The TDMA-based communication pattern is established by a superframe structure, which defines the transmission behaviors in a sequence of consecutive time slots. In RT-WiFiQA, each transmission in the superframe is configured with a link type (i.e., downlink or uplink), an index of the targeted destination, a transmission rate, MAC-layer retransmission times, and an RT-QoS indicator. The maximum MAC-layer retransmission times can be evaluated through the transmission rate, allocated time slots, and packet lengths. The network profile plays the role of the bridge between the APP layer and the MAC layer and can be generated through our APIs. The scheduler on the MAC layer follows the superframe structure to transmit packets with transmission rates and retransmission times.

The RT-QoS setting is attached with each packet to distinguish different traffic classes, which have different transmission patterns on the MAC layer. We design APIs for RT-QoS based on the existing type of service (ToS) field in the IP header. Users can determine a ToS value for each frame classified to a specific Access Categories (AC) value on the MAC layer. According to the different AC values, we design a first-in-first-out (FIFO) queue system for each RT-QoS traffic. We create three RT-QoS traffic types as follows:

- *RTB*: Real-time data transmissions through broadcast. The packets stored in the RTB queue can use the FGA mechanism, which aggregates the RTB packets and broadcasts the aggregated packet to multiple desired stations. The RTB queue is particularly suitable for industrial downlink data transmissions using the UDP protocol where ACKs are not required from the MAC layer. In our protocol design, the users can determine whether to use the aggregation by enabling the FGA and choosing the RT-QoS through our APIs.
- *RTU*: Real-time transmissions through unicast. Packets stored in the RTU queue will not use the FGA mechanism and are transmitted according to the scheduler by unicasting. The RTU queue is compatible with all the existing upper-layer protocols, e.g., UDP and TCP, for both uplink and downlink. If there is no RTU packet buffered, RTB packets are allowed to be transmitted in unicast within the time slot allocated to RTU.
- *NRT*: Non-real-time transmissions. NRT packets are transmitted only within a temporal window, namely NRT window (NRTW). The transmission of NRT packets will strictly not exceed NRTW and influence the real-time traffic. The NRT queue is also compatible with all the existing upper-layer protocols and suitable for packets without latency requirements.

**Figure 2:**
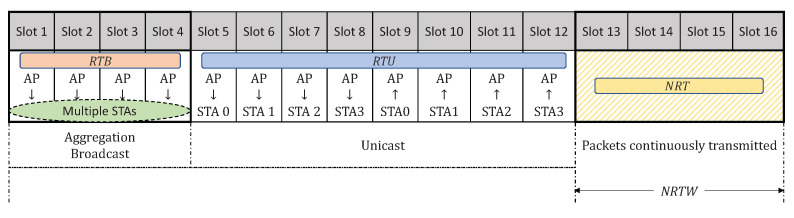
An example of the RT-WiFiQA superframe design.

Figure 2 is an example of the superframe structure. There is an RT-QoS indicator defined for each time slot to determine the packet selection from different queues. The first four time slots are used for RTB packets, which can be transmitted through multiple time slots because of the FGA mechanism. Slots 5 to 12 are reserved for RTU packets, including both uplink and downlink. NRTW for NRT packets is from the 13th slot to the last. In the NRTW, we adopt a best-effort transmission scheme to transmit as many packets as we can under the constraint that the packet transmissions do not exceed the NRTW. In the best-effort scheme, we first fetch a packet from the NRT queue and estimate its transmission duration according to its packet length, transmission rate, and retransmission times. The transmitter can reserve the duration for each packet and trigger the next transmission after this duration time. This process keeps executing until the end of the NRTW.

### 3.2. FGA

The FGA process starts with the selection of packets for aggregation. Once the timer triggers a transmission, the scheduler first enables the FGA if the RT-QoS setting of the current time slot is RTB. Then, the scheduler continues to search for packets in the RTB queue and considers both the packet length and critical threshold of each packet to determine whether a new packet can be transmitted through the FGA mechanism. The critical threshold can be evaluated according to Section 4.1. Specifically, we need to make sure the aggregated packet length is smaller than the critical threshold of the new packet and each selected packet for aggregation. Otherwise, the new packet cannot be transmitted through FGA. The scheduler will first fetch packets for different stations according to the scheduling information of the superframe. When the scheduler cannot find such packets from the RTB queue and the aggregated packet length is smaller than the critical threshold, it will seek other existing UDP packets from all RT-QoS queues for aggregation, following the sequence of RTB, RTU, and NRT, until the critical length constraint is reached. This is because our FGA scheme is compatible with all UDP packets. Additionally, if no packet can be aggregated to the head-of-line packet, the scheduler will transmit the packet without FGA by using one time slot. In the next time slot, the scheduler continues to fetch the new head-of-line packet in the RTB queue and search for other packets that can be aggregated. This procedure keeps executing until reaching the end of the allocated time slots for the RTB transmission.

**Figure 3:**
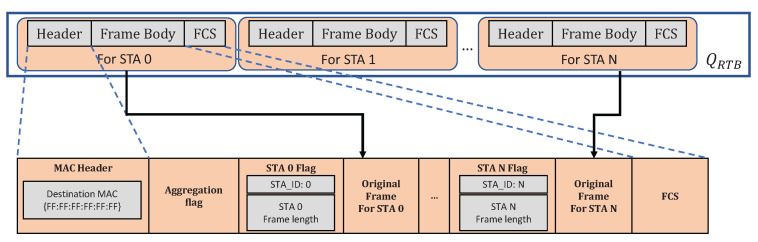
Typical FGA frame format.

After the selection of FGA packets, the scheduler will initially push these packets into a temporal singly linked list. The first frame in the linked list will keep slices of the MAC header and frame check sequence (FCS). A one-byte hexadecimal aggregation flag is appended after the MAC header for recognition of the aggregated packet. Then, a unique station flag will be generated for each frame, including the station identity recorded in the device table and a frame length. The scheduler further appends the combinations of one station flag and its corresponding full original frame after the aggregation flag. Finally, the destination address at the MAC header of the aggregated frame is changed to the broadcast MAC address, e.g., 0xFF. An example of the final aggregated frame format is shown in Figure 3.

The disaggregation process occurs when a station receives the aggregated frame. The station determines whether the packet is sent through the aggregation process based on two conditions: (1) the destination MAC address is a broadcast address; (2) the octet after the MAC header is the special aggregation flag. Then, the receiver reads the aggregated frame from the aggregation flag to the end of the frame. Once the station identity in the station flag is matched with its own identity, the station will keep the followed frame with the frame length recorded in the station flag, and the rest parts of the aggregated frame will be discarded. If the station identity is not matched, the station will skip the frame length recorded in the station flag and read the next station flag until the end pointer of the aggregated frame.

## 4. FGA Analysis and Numerical Results

With a given time budget for transmissions, i.e., the number of allocated time slots, aggregation of multiple packets can potentially retransmit more times than the conventional non-aggregated transmission. It is because aggregation reduces the overhead of the PHY layer significantly, especially when the payload size of the packets is relatively small. However, aggregation leads to a larger packet size, which may increase the PER of the transmitted packet compared with the non-aggregated transmission. Therefore, it is important to determine whether the aggregation mechanism will benefit the system in terms of reliability and latency. Moreover, how shall we choose the system parameters for the packet length of the aggregated packet in order to achieve higher reliability with the bounded latency requirement? To answer these critical questions, in the following, we compare the reliability performance of aggregation and non-aggregation schemes under a predefined latency constraint, i.e., a given number of allocated time slots.

### 4.1. Trade-Off Analysis of FGA

We assume that a total number of *n* packets can be aggregated for transmission. For a fair comparison, each packet without using aggregation will occupy one time slot according to the existing non-aggregation mechanisms. Differently, the aggregated *n* packets can utilize *n* time slots for transmission such that the total time consumption for the non-aggregation and aggregation schemes are the same. Note that the latency constraint considered in this case is *n* time slots for all the packets. It is reasonable because the slot duration of the TDMA-based system is very small, e.g., in the level of μs, and users can choose the setup of *n* depending on the specific application requirements. Let $l_{i},1\leq i\leq n$ denote the length of *i*th packet and $l_{a,i}$ denote the total length of packets that can be aggregated to the *i*th packet. $T_{s}$ denotes the duration of one time slot, and *r* denotes the transmission rate. We define $T_{i}$ as the transmission time of the *i*th packet without using FGA, and $T_{a,i}$ as the transmission time of the *i*th packet adopting FGA with a total packet length of $l_{i}+l_{a,i}$. Based on $l_{i}$ and $l_{a,i}$, we can derive that

(1) $$\begin{array}{cc} \; & T_{i}=\frac{l_{i}}{r}+T_{PLCP}+T_{DIFS}, \end{array}$$

(2) $$\begin{array}{cc} \; & T_{a,i}=\frac{l_{a,i}+l_{i}}{r}+T_{PLCP}+T_{DIFS}, \end{array}$$

where $T_{PLCP}=20\;\mu$s is the physical layer convergence procedure (PLCP) preamble with header delay of each packet transmitted by the IEEE 802.11 PHY layer [24]; $T_{DIFS}$ = 28 μs is the inter-frame spacing. We now define the maximum transmission times for the *i*th packet without using FGA as $M_{i}$ and that for the *i*th packet adopting FGA as $M_{a,i}$. They can be calculated as

(3) $$\begin{array}{cc} \; & M_{i}=\frac{T_{s}-T_{g}}{T_{i}}, \end{array}$$

(4) $$\begin{array}{cc} \; & M_{a,i}=\frac{nT_{s}-T_{g}}{T_{a,i}}, \end{array}$$

where $T_{g}=20\;\mu$s is the guard time to tolerate the synchronization error.

To verify whether our FGA scheme can improve the reliability, we compare the PER performance of the FGA with the conventional non-aggregation scheme. The PER is defined as the probability that a packet cannot be successfully transmitted after all transmissions and retransmissions within the same amount of allocated time slots. To capture the PHY-layer overhead, we model the PLCP preamble data length as $l_{o}=rT_{PLCP}$ because the PLCP preamble is transmitted at 1 Mbps [25]. Based on the above model, we define $P_{i}$ as the PER of the *i*th packet without using FGA and $P_{a,i}$ as the PER of the *i*th packet adopting FGA. Let *p* denote the bit error rate (BER). $P_{i}$ and $P_{a,i}$ can be evaluated by

(5) $$\begin{array}{cc} \; & P_{i}={\left(1-{\left(1-p\right)}^{l_{i}+l_{o}}\right)}^{M_{i}}, \end{array}$$

(6) $$\begin{array}{cc} \; & P_{a,i}={\left(1-{\left(1-p\right)}^{l_{i}+l_{a,i}+l_{o}}\right)}^{M_{a,i}}. \end{array}$$

The value of *p* can be evaluated approximately by the long-term average PER at each station using Equations (5) and (6), which can be acquired in an offline manner.

To compare the PER of non-aggregation and aggregation, in the following, we mathematically derive the solution for $P_{a,i}\leq P_{i}$, such that the FGA scheme can outperform the non-aggregation transmission. Because $P_{i},P_{a,i}>0$, we first take the logarithm on both sides of the inequality. We then define $f\left(l_{a,i}\right)=\ln\left(P_{a,i}\right)-\ln\left(P_{i}\right)$. The inequality $P_{a,i}\leq P_{i}$ is thus equivalent to

(7) $$\begin{array}{cc} \; & f\left(l_{a,i}\right)=\ln\left(P_{a,i}\right)-\ln\left(P_{i}\right)\leq 0. \end{array}$$

The solution to the inequality can be summarized and given in Proposition 1.

**Proposition** **1.** There exists a unique solution $l_{a,i}^{*}$ to Equation (7), such that when $l_{a,i}\leq l_{a,i}^{*}$, $P_{a,i}\leq P_{i}$, and the FGA scheme outperforms non-aggregation in terms of reliability. Otherwise, $P_{a,i}>P_{i}$, and the FGA scheme has a higher PER.

**Proof.**  We first prove that function $f(l_{a,i})$ is a monotonically increasing function of $l_{a,i}$ and then prove there exists a unique solution for Equation (7). Let $T_{o}=T_{PLCP}+T_{DIFS}$, and Equation (7) can be simplified to

(8) $$\begin{array}{cc} f(l_{a,i}) & =\frac{nT_{s}-T_{g}}{\frac{l_{i}+l_{a,i}}{r}+T_{o}}\ln\left(1-{\left(1-p\right)}^{l_{i}+l_{o}+l_{a,i}}\right)-\\ \; & \frac{T_{s}-T_{g}}{\frac{l_{i}}{r}+T_{o}}\ln\left(1-{\left(1-p\right)}^{l_{i}+l_{o}}\right). \end{array}$$

To prove the monotonicity, the first-order derivative of Equation (8) with respect to $l_{a,i}$ can be evaluated by

(9) $$\begin{array}{cc} \frac{df}{dl_{a,i}} & =-\left(nT_{s}-T_{g}\right)\left(\frac{r\ln\left(1-{\left(1-p\right)}^{l_{i}+l_{o}+l_{a,i}}\right)}{{\left(l_{i}+l_{a,i}+rT_{o}\right)}^{2}}+\right.\\ \; & \left.\frac{r{\left(1-p\right)}^{l_{i}+l_{o}+l_{a,i}}\ln\left(1-p\right)}{\left(1-{\left(1-p\right)}^{l_{i}+l_{o}+l_{a,i}}\right)\left(l_{i}+l_{a,i}+rT_{o}\right)}\right)>0. \end{array}$$

Due to 0<p<1, it can be readily verified that the above inequality holds. Therefore, Equation (8) is monotonically increasing with $l_{a,i}$.

We now prove there exists a unique solution of Equation (8). On one hand, because $l_{a,i}\geq 0$, we have

(10) $$f\left(0\right)=\frac{\left(n-1\right)T_{s}}{\frac{l_{i}}{r}+T_{o}}\ln\left(1-{\left(1-p\right)}^{l_{i}+l_{o}}\right).$$

Due to n≥1, it can be verified that f(0)≤0. On the other hand, if $l_{a,i}$ approach infinity, we have

(11) $$f\left(+\infty \right)=-\frac{T_{s}-T_{g}}{\frac{l_{i}}{r}+T_{o}}\ln\left(1-{\left(1-p\right)}^{l_{i}+l_{o}}\right)>0.$$

With the monotonicity of $f(l_{a,i})$, we can deduce that there must exist a unique solution $l_{a,i}^{*}$ such that $f(l_{a,i}^{*})=0$. This completes the proof. □

Based on the Proposition 1, we can obtain the critical aggregated packet length $l_{a,i}^{*}$ for each packet length $l_{i}$ by solving $f(l_{a,i})=0$. Due to the complicated structure of $f(l_{a,i})$, it is intractable to obtain a closed-form expression of $l_{a,i}^{*}$. Fortunately, $l_{a,i}^{*}$ can be solved through numerical methods such as the bisection method. To determine whether the *i*th packet can be aggregated, packets from the first to the *i*th need to meet their individual requirement of the critical length threshold. Specifically, the *i*th packet can be aggregated only if for ∀j∈[1,i], $l_{a,j}\leq l_{a,j}^{*}$. Based on the above analysis, we can then determine whether a packet should be transmitted through the FGA scheme or unicast to achieve optimal reliability in the RT-WiFiQA system.

### 4.2. Numerical Results

**Figure 4:**
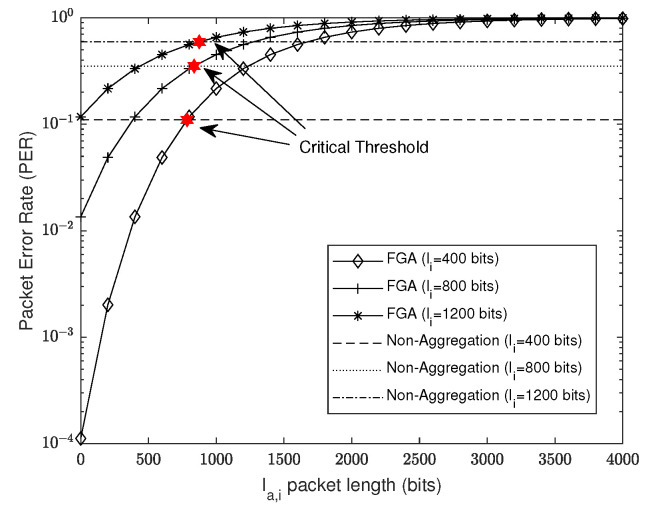
PER against aggregated packet size $l_{a,i}$ for different $l_{i}$, where the critical thresholds can be evaluated based on Proposition 1.

We consider a setup with four time slots allocated for RTB transmissions with a time slot of 512 μs, and a BER of $1.3\times 10^{-3}$ for the link, which is comparable with our practical setup in Section 5.1. In Figure 4, we depict the PER against $l_{a,i}$ for different $l_{i}$ and compare the performance of FGA with the non-aggregation scheme. The PER of the non-aggregation scheme does not change with $l_{a,i}$ and is shown as a horizontal line, while the curves of FGA are increasing as $l_{a,i}$ grows because the PER of FGA is monotonically increasing with $l_{a,i}$, as discussed in Section 4.1. We also depict the critical threshold of the FGA scheme according to Proposition 1 by using a bisection method. From Figure 4, we can observe that each FGA curve and the non-aggregation line have a unique intersection point, which coincides with our theoretical critical threshold. If $l_{a,i}$ is smaller than the critical threshold, the FGA scheme can have a lower PER than the non-aggregation scheme, and vice versa. It validates our analysis provided in Section 4.1.

**Figure 5:**
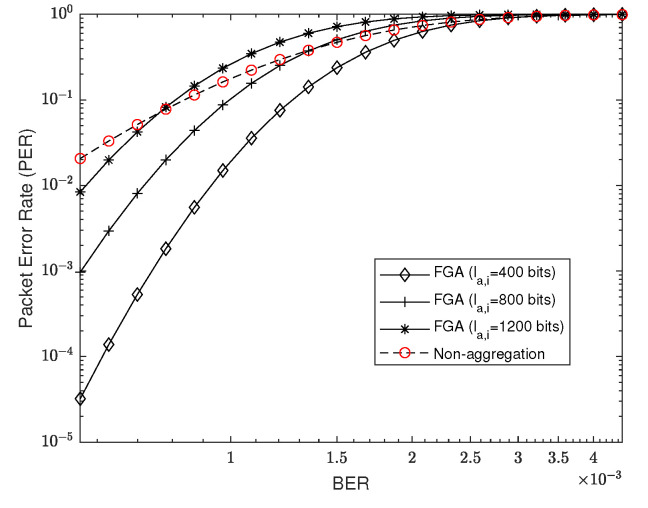
PER against increasing BER for different total length $l_{a,i}$ of packets that can be aggregated to the *i*th packet with $l_{i}$ of 800 bits.

We then show the PER against different BER *p* in Figure 5 and set $l_{i}$ as 800 bits. In Figure 5, we can first observe that the PER curves grow as BER increases. If the BER is relatively low, the FGA scheme can achieve a lower PER compared with the non-aggregation scheme for a large variety of $l_{a,i}$. Otherwise, the FGA scheme can achieve a lower PER only for a small $l_{a,i}$. This observation indicates that packets with a large size can be aggregated when the channel condition has a mild BER. It is because a good channel condition can achieve a low PER even when the packet size is large.

## 5. Experiments and Results

**Figure 6:**
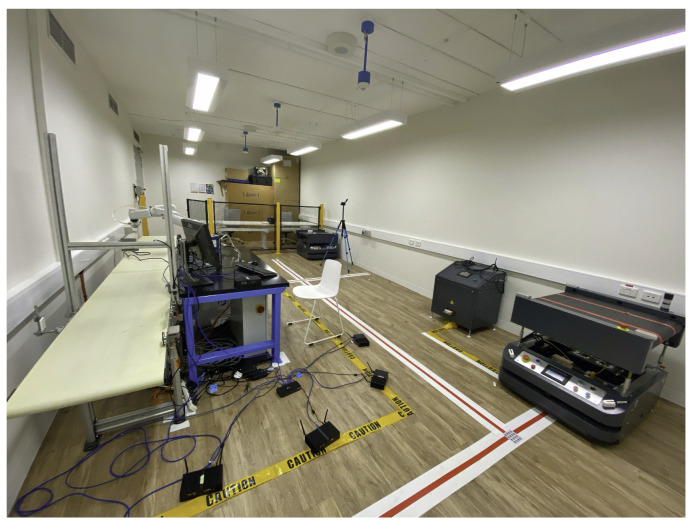
Experiment platform.

Our experiment design and performance evaluation are presented in this section. In our experimental platform, we use miniPCs from Qotom [26] for AP and stations. The miniPCs run a Ubuntu 14.04 operating system, and the Linux kernel version is 3.13.0-32. The CPU used for AP is Intel Core i5-4200U, while the one for stations is Intel Core i3-5005U. The RAM of all devices is 8G. For the IEEE 802.11 interface, we choose the Atheros NIC AR9285, which supports IEEE 802.11 b/g/n protocols and uses open-source driver ATH9k [27]. Besides one AP and four stations, an additional PC is used to monitor and evaluate the performance of all devices. Figure 6 presents our experimental platform, which is comparable with the practical IIoT network. In order to obtain practical results in a real channel environment, we implemented the proposed schemes on our platform using 802.11 b/g/n PHY layer due to the available open-source driver. Nevertheless, our protocol design can also be extended to more advanced IEEE 802.11 ac/ax with orthogonal frequency-division multiple access (OFDMA) PHY layer by further improving the time-domain transmission efficiency and reliability within a given number of resource units, which will be left as future work.

### 5.1. Experiment Design

We measure the performance on both the MAC layer and the APP layer, and the performance metrics include latency and reliability. The MAC-layer results aim to validate the bounded latency and optimized reliability performance of the proposed RT-WiFiQA protocol compared with WiFi and RT-WiFi. The APP-layer results can present the overall transmission latency and reliability of IIoT applications because packets are processed to the APP layer ultimately. Specifically, the MAC-layer latency is the time difference between one packet leaving the transmitter’s MAC layer and entering the receiver’s MAC layer. The MAC-layer reliability is measured by the ratio of successfully received packets to the total number of transmitted packets on the MAC layer. The APP-layer latency is the time difference between one packet generated by the transmitter application and successfully received by the receiver application. The APP-layer reliability is defined as the ratio of successfully received packets by the receiver application to the total number of packets transmitted from the transmitter application. For the measurement of latency in the MAC and APP layers, the synchronization between the MAC layer is realized by the timing synchronization function (TSF) of Linux [5] with a drift lower than 20 μs. The synchronization between the applications of transmitter and receiver is achieved by IEEE 1588 Precision Time Protocol (PTP) [28] with its software tool PTP daemon (ptpd) [29]. The APP-layer synchronization error is smaller than 40 μs. The synchronization error is acceptable for our delay measurement, where the MAC-layer delay is around 300 μs and the APP-layer delay is on the level of ms.

Our experiments focus on the downlink performance of our proposed mechanisms. In the following experiments, we compare the MAC-layer and the APP-layer performance of RT-WiFiQA with RT-WiFi and conventional WiFi. RT-WiFi is a basic TDMA system based on 802.11 interfaces without considering the proposed RT-QoS and FGA mechanisms. We develop applications in Python 3 to simulate downlink traffic with different RT-QoS types as well as uplink traffic. The different programs can reveal the concurrent running state of multiple practical applications with various workloads and QoS requirements. All of the applications generate packets of different types with a length of 50 bytes every 20 ms, which are represented as the traffic payload and packet interval in Table 1. We execute a combination of the three applications for each station concurrently, and each experiment lasts 40 min. Note that we add uplink traffic to our experiments, but the uplink performance of the proposed RT-WiFiQA is similar to the result of RT-WiFi, which was extensively investigated in [5]. We also measure the approximate average BER of the four stations by measuring the long-term average PER using Equations (5) and (6). According to the offline measurement, we set $p=1.3\times 10^{-3}$ for all the considered stations, which is used to determine the critical thresholds for each station in our proposed FGA algorithm. A time slot duration of 512 μs and a PHY-layer transmission rate of 36 Mbps are set to both RT-WiFi and RT-WiFiQA. The MAC-layer retransmission times are pre-calculated according to Equations (3) and (4), which are four times for transmissions without using FGA and ten times for our FGA packets. The APP-Re is set to four times in terms of the COTS configuration.

### 5.2. MAC-Layer Performance

**Figure 7:**
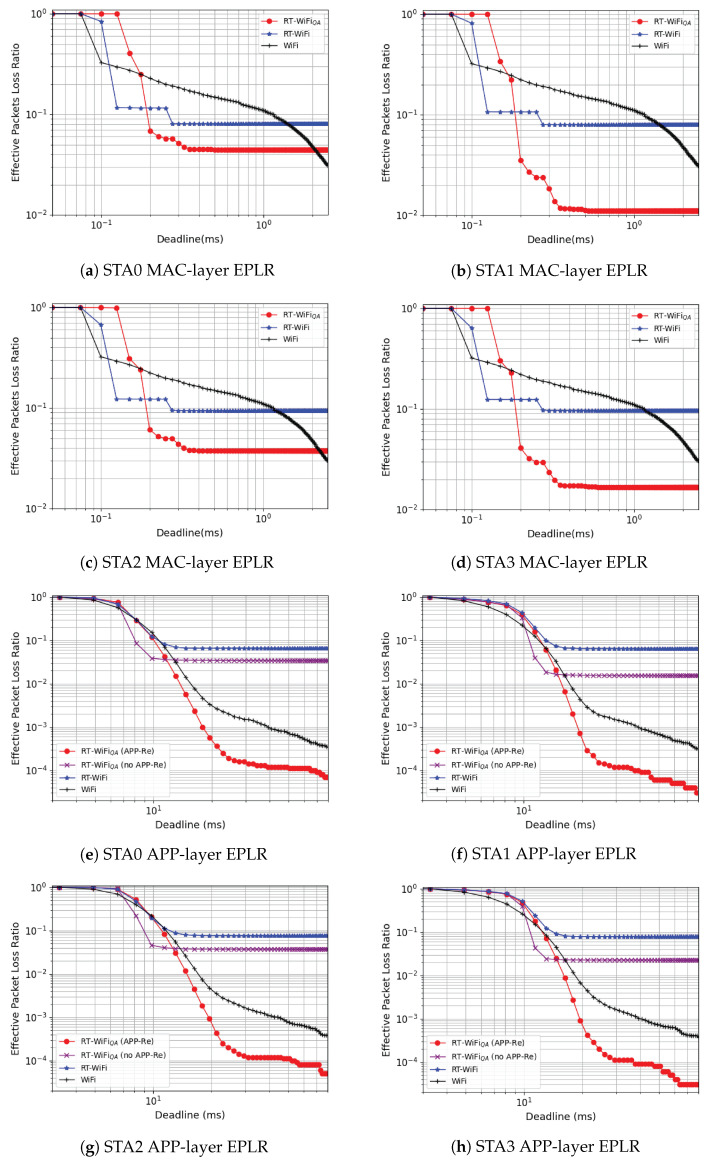
CCDF curves of RT-WiFiQA EPLR performance in terms of the deadline requirements, compared with RT-WiFi and WiFi. Figure 7a–d show the MAC-layer EPLR, and Figure 7e–h show the APP-layer EPLR.

We define the deadline as the required latency performance of a given application, and the effective packet loss ratio (EPLR) as the percentage of packets unsuccessfully received at or exceeding the given deadline. We use complementary cumulative distribution function (CCDF) curves in Figure 7 to present the EPLR performance of all the stations, which can indicate the trade-off between latency and reliability. We first plot the MAC-layer EPLR performance in Figure 7a–d. The curves of RT-WiFi and WiFi drop earlier than RT-WiFiQA, illustrating that the minimum achievable delay of RT-WiFi and WiFi is lower than RT-WiFiQA. It is because the proposed FGA mechanism leads to a larger packet size such that the FGA packets cannot be transmitted within an extremely small amount of time. The downward tendency of RT-WiFiQA and RT-WiFi is concentrated upon the mean delay and follow a step case. Differently, the curve of WiFi shows a gradual downward trend. It is because the WiFi system uses a dynamic rate control algorithm, i.e., Minstrel [30], but RT-WiFi and RT-WiFiQA choose a fixed rate and retransmission times setting. This observation also presents the proposed RT-WiFiQA and RT-WiFi can guarantee bounded latency. Moreover, RT-WiFiQA achieves lower EPLR than RT-WiFi. It is because the proposed FGA scheme can potentially increase the reliability of the system by reducing the transmission overhead and allow for more retransmission times. Lastly, WiFi can outperform RT-WiFi and RT-WiFiQA when the delay requirement is very high, e.g., more than 2 ms for STA0. It is because WiFi uses the CSMA/CA scheme and has a larger number of retransmissions. Differently, RT-WiFi and RT-WiFiQA apply limited retransmission times in order to achieve bounded latency.

### 5.3. APP-Layer Performance

We now turn to the APP-layer EPLR performance and show the CCDF curves in Figure 7e–h. For a more comprehensive comparison, we also add a benchmark curve of RT-WiFiQA without the APP-Re. Taking STA1 as an instance, the curve of WiFi starts to drop first; maintains a gradual downward tendency as the delay grows; and outperforms other systems when the delay is relatively small, e.g., from 5 ms to 10 ms. It is also because WiFi uses the Minstrel rate control algorithm, which may select higher rates than the fixed rate used in the TDMA systems. RT-WiFiQA without APP-Re performs best as the delay requirement is from 10 ms to 15 ms. Compared with RT-WiFi, RT-WiFiQA with the RT-QoS scheme can transmit real-time packets without internal interference from non-real-time packets, leading to smaller APP-layer latency. Compared with WiFi, due to the coexistence of both uplink and NRT traffic, the CSMA/CA mechanism of WiFi leads to a longer back-off delay. When the delay requirement is over 12 ms, RT-WiFiQA without APP-Re and RT-WiFi almost reach the bound of their reliability, and RT-WiFiQA with APP-Re performs the best because the APP-Re can effectively combat the burst interference. At the delay of 40 ms, RT-WiFiQA can ultimately achieve an EPLR of $10^{-4}$. The achievable latency and reliability on the APP layer can benefit many existing IIoT applications, such as the wireless control of Automated Guided Vehicles (AGVs) for logistic sorting [23,31], and the interlocking control systems in process automation domain [32].

## 6. Conclusions and Future Work

In this paper, we develop the RT-WiFiQA protocol with two novel schemes, i.e., RT-QoS and FGA, for IIoT applications based on 802.11 TDMA systems. The RT-QoS protocol is used to guarantee the latency and reliability performance of real-time traffic when multiple types of traffic coexist and to support the proposed FGA mechanism. The FGA mechanism can aggregate multiple packets for different stations and reduce the transmission overhead to improve the efficiency and reliability of the system. We aim to provide a flexible design by developing APIs for configuration of RT-WiFiQA and to provide insights on network parameter selection. Based on the observation of the trade-off between the FGA packet size and reliability, we analytically derive a critical threshold such that the FGA scheme can outperform non-aggregation in terms of reliability when the aggregated packet size is smaller than the critical threshold and provide numerical results. We also implement the proposed RT-WiFiQA protocol on the COTS hardware running the Linux system and conduct extensive experiments to compare the performance of RT-WiFiQA with RT-WiFi and conventional WiFi. The experiment results demonstrate that RT-WiFiQA can promise higher reliability than RT-WiFi and guarantee a real-time performance compared with WiFi on both the MAC and APP layers.

Despite the fact that our proposed RT-WiFiQA protocol can improve the latency and reliability performance compared with the RT-WiFi and legacy WiFi, it still has some limitations that need to be addressed in the future. First, the achievable reliability of the designed RT-WiFiQA protocol is confined by our designed rate control mechanism, where a fixed rate is adopted for each packet transmission. To further improve the performance, in our future work, we will develop advanced rate control mechanisms by using machine learning algorithms for the proposed RT-WiFiQA protocol that can select the rate adaptively according to the dynamics of the channel environment. Second, the current RT-WiFiQA protocol is designed and implemented on 802.11 b/g/n COTS chips where an OFDM physical layer is adopted. Due to the hardware limitations, we are not able to extend them to the recent OFDMA 802.11 systems, e.g., 802.11ax. It is important to redesign the proposed protocol for OFDMA systems and to evaluate its performance on OFDMA systems, which will be left as our future work.

There are multiple interesting topics to be explored in our future work. First, we optimize the FGA scheme on the more recent 802.11ax and 802.11be interfaces. Our current FGA scheme focuses on the resource allocation in the time domain for 802.11 b/g/n. Note that in the OFDMA systems, the resource allocation needs to consider both the time-domain and frequency-domain resources by allocating the resource units to different devices. In this way, the FGA algorithm needs to be further optimized for more efficient transmissions, and the trade-off analysis provided in this paper needs to be revisited. Moreover, to combat the random burst interference and to improve the reliability performance, adaptive rate control mechanisms need to be developed to deal with the dynamics of the wireless environment. Other critical features such as throughput, energy efficiency, and security should be considered in future work.

## Figures and Tables

**Table 1:** Experimental parameters preset for RT-WiFiQA.

Configuration	Parameter
ine Number of stations	4
MAC retransmissions	10
Traffic payload (bytes)	50
Packet interval (ms)	20
Transmit rate (Mbps)	36
Time slot (μs)	512
Test duration per group (min)	40

## Data Availability

Data sharing not applicable.
